# Inclusion of gametocyte parameters in anti-malarial drug efficacy studies: filling a neglected gap needed for malaria elimination

**DOI:** 10.1186/s12936-015-0936-4

**Published:** 2015-10-19

**Authors:** Rashad Abdul-Ghani, Leonardo K. Basco, John C. Beier, Mohammed A. K. Mahdy

**Affiliations:** Department of Parasitology, Faculty of Medicine and Health Sciences, Sana’a University, Sana’a, Yemen; Tropical Disease Research Center, University of Science and Technology, Sana’a, Yemen; Unité de Recherche 198, Unité de Recherche sur les Maladies Infectieuses et Tropicales Emergentes, Institut de Recherche pour le Développement, Faculté de Médecine La Timone, Aix-Marseille Université, Marseille, France; Department of Public Health Sciences, University of Miami Miller School of Medicine, Miami, FL USA

**Keywords:** *Plasmodium*, Gametocyte, Anti-malarial drug, Drug resistance, Efficacy study, Malaria elimination

## Abstract

Standard anti-malarial drug efficacy and drug resistance assessments neglect the gametocyte parameters in their protocols. With the spread of drug resistance and the absence of clinically proven vaccines, the use of gametocytocidal drugs or drug combinations with transmission-blocking activity is a high priority for malaria control and elimination. However, the limited repertoire of gametocytocidal drugs and induction of gametocytogenesis after treatment with certain anti-malarial drugs necessitate both regular monitoring of gametocytocidal activities of anti-malarial drugs in clinical use and the effectiveness of candidate gametocytocidal agents. Therefore, updating current protocols of anti-malarial drug efficacy is needed to reflect the effects of anti-malarial drugs or drug combinations on gametocyte carriage and gametocyte density along with asexual parasite density. Developing protocols of anti-malarial drug efficacy that include gametocyte parameters related to both microscopic and submicroscopic gametocytaemias is important if drugs or drug combinations are to be strategically used in transmission-blocking interventions in the context of malaria elimination. The present piece of opinion highlights the challenges in gametocyte detection and follow-up and discuss the need for including the gametocyte parameter in anti-malarial efficacy studies.

## Background

There are several integrated approaches for the assessment of anti-malarial drug efficacy and drug resistance: therapeutic efficacy in symptomatic patients with uncomplicated malaria, in vitro*/*ex vivo assays, molecular markers of drug resistance, and measurement of plasma concentration of anti-malarial drugs [1, 2]. Clinical studies are the gold standard for assessing the efficacy of anti-malarial drugs, and their outcomes represent the primary data used for making malaria treatment policies by national and regional control programmes [3, 4]. Apart from the clinical assessment of therapeutic response to the drug or drug combinations, the parasitological parameters adopted in different protocols of clinical studies, including the standard World Health Organization (WHO) protocols, only address the decrease in parasite density of asexual forms [5–8]. Parasitologically, therefore, drug efficacy studies do not consider the drug effect on gametocyte carriage and density but focus on asexual parasite density. This is attributed to the fact that clinical presentation of malaria is a direct consequence of the asexual erythrocytic stages and that gametocytes are not responsible for the clinical disease. Although these protocols are useful to measure the schizonticidal efficacy, they do not consider the gametocytocidal activity of the tested anti-malarial drug or drug combinations. The question remains as to why the gametocyte parameter is not routinely investigated during the 28–42 day follow-up period. The gametocytocidal action and transmission-blocking potential are not the major issues in most drug efficacy studies that primarily consider the schizonticidal action of anti-malarial drugs. Several clinical studies have focused on gametocytocidal action of different drugs [9–11], but once it is established, most researchers may not feel the need to re-assess gametocytocidal efficacy of those drugs.

At present, 8-aminoquinolines (primaquine and tafenoquine) are the only gametocytocides that are highly effective against gametocytes of all human malaria species. Artemisinin derivatives do not kill mature gametocytes of *Plasmodium falciparum*, but indirectly inhibit gametocyte development by killing immature gametocytes and rapidly killing asexual erythrocytic parasites before gametocytogenesis occurs [12]. Although primaquine has been shown to stop transmission before the disappearance of gametocytes from the peripheral blood on microscopy, its use as a gametocytocidal drug is associated with some risks, such as haemolytic toxicity among patients with glucose-6-phosphate dehydrogenase deficiency which occurs at high frequencies (3–30 %) in some endemic countries, hindering its use on a large scale in elimination strategies [13–15]. In addition, alternative gametocytocidal drugs, such as tafenoquine, are still under clinical investigation. Recently, Eziefula et al. [14] reported the lack of informativeness of gametocyte data in primaquine trials and proposed a strategic plan to optimize trial design for efficient interruption of malaria transmission. These authors also recommended the establishment of standard protocols to assess the efficacy of novel gametocytocidal agents, such as tafenoquine and methylene blue. This supports the present opinion piece to include the gametocyte parameter in standardized protocols of regular anti-malarial monitoring studies.

## Is it necessary to include the gametocyte parameter in anti-malarial drug efficacy studies?

Even if the drug is effective in clearing asexual parasitaemia and resolving malaria-associated signs and symptoms, gametocytogenesis can be initiated after the use of certain anti-malarial drugs, such as chloroquine and sulfadoxine-pyrimethamine [16]. Given that anti-malarial drug resistance is widespread and drug-resistant parasites are more likely to produce large numbers of gametocytes, compared to drug-sensitive parasites, the transmissibility of gametocytes carrying drug-resistant alleles is enhanced [10, 17–24]. The inclusion of gametocyte parameters in the standard tests used to regularly assess anti-malarial drug efficacy in such situations would be of great value to deter anti-malarial drug resistance.

To attain malaria elimination, anti-malarial drugs or drug combinations should not only clear the asexual parasite stages responsible for clinical disease but also clear the sexual stages that maintain its transmission. To address the need for the strategic use of anti-malarial drugs in blocking malaria parasite transmission, the parallel inclusion of the gametocyte parameter with asexual parasite density should be prioritized in drug efficacy studies [12, 16]. Anti-malarial chemotherapy should specifically target gametocyte clearance to reduce or block parasite transmission and to prevent selection and spread of resistant strains [16, 25, 26].

It remains controversial whether the establishment in a clinical study that a certain anti-malarial drug has a gametocytocidal action is enough, ignoring the assessment of such action in subsequent efficacy studies. Several considerations suggest the necessity to regularly assess the gametocytocidal action of anti-malarial drugs in efficacy studies. First, the complexity of gametocytogenesis that involves the interaction of several host, environmental and biological factors [27, 28], including treatment with certain anti-malarial drugs, collectively leads to variability in gametocyte carriage and density. For instance, the gametocytocidal action of a drug in a certain geographical area could be different from another depending on certain factors such as the level of malaria endemicity and the immune status of exposed population. Second, there could be a seasonal variation in the rate of gametocytaemia in the same area [29], which requires further investigation of possible variations in the gametocytocidal action of anti-malarial drugs intended to interrupt malaria transmission in that area. Third, in the face of increasing resistance to anti-malarial drugs that favour the increase in gametocyte density and/or prevalence [30, 31], a regular monitoring of gametocytocidal activity of the drug in relation to the resistance situation to a drug becomes a priority to block parasite transmission and spread of drug resistance. A major stride in this respect is the ongoing analysis of *P. falciparum* gametocyte carriage after treatment with different artemisinin-based combination therapy (ACT) from regions with different endemicity, which is being carried out by the Gametocyte Carriage Study Group of the Worldwide Anti-malarial Resistance Network [32]. This initiative undertakes the determination of gametocyte density or prevalence at enrolment and during follow-up as an inclusion criterion. The main focus is on epidemiological factors associated with gametocyte carriage and clearance after treatment with different ACT across different countries. Accordingly, it will likely contribute to devising standardized protocols for the assessment of anti-malarial drug and drug combination efficacy that include the effects of different drug regimens on gametocyte carriage rate and duration within regular activities of national and regional malaria control programmes.

## Challenges and prospects in gametocyte detection and follow-up

One of the major challenges is the gap between the detectable gametocytes and infective level of gametocytaemia. Although the microscopic detection of *Plasmodium* gametocytes may be as low as 10–20 gametocytes/µl [33], sub-microscopic gametocyte densities can infect vector mosquitoes [34–36]. In the standard WHO protocol for therapeutic efficacy, the microscopist reports whether gametocytes were observed while performing routine counts of asexual parasites, which is easier for the characteristic banana-shaped *P. falciparum* gametocytes compared to those of other species (which are rounded bodies that may be mistaken for mature asexual trophozoites). Sensitivity of microscopy is not enough for the detection of a low gametocytaemia [37]. In addition to being unrelated to clinical signs and symptoms of the disease, the longer time required to detect and count gametocytes is one of the major reasons to ignore routine counting of gametocytes during anti-malarial efficacy trials. Two types of counts are suggested to be performed on thick blood smears: a first count of asexual parasites against 200 white blood cells (WBCs) according to standard WHO protocols and a second count for gametocytes against at least 500 WBCs as previously reported [38, 39]. However, counting gametocytes against 500 WBCs should be revised and optimized as this comes down to about 20–30 high power fields, which seems to be far too little.

The question remains as for how long the presence of gametocytes should be monitored after treatment, i.e., the duration of gametocyte carriage. The follow-up intervals for the assessment of gametocyte carriage and gametocyte density should be different from those of asexual parasites because of the slower development of *P. falciparum* gametocytes (up to 10 days) compared to that of asexual erythrocytic stages [40]. In general, gametocytaemia is usually measured on a weekly basis starting on the day of treatment until the end of the fourth week of treatment when it peaks and then starts to decline [22]. Therefore, there is a need to establish the best chronological follow-up pattern that would even extend beyond that for asexual parasitaemia due to the relative persistence of gametocytes in the blood. The period of follow-up will be different, where asexual parasites should be counted according to standard WHO protocols (i.e., 28–42 days) whereas gametocytes should be screened at weekly intervals for a longer period, at least 42 days. However, optimization of the follow-up period for gametocytes is yet to be explored. It is noteworthy that an innovative magnetic fractionation approach for on-site counting of gametocytes in field studies has been introduced as an alternative to light microscopy with a sensitivity comparable to that of reverse transcriptase polymerase chain reaction (RT-PCR) [39].

In case of sub-microscopic infections where counts could not be performed, gametocytes can be detected and their load can be quantified using molecular techniques. The adoption of specific molecular approaches, such as real-time RT-PCR for gametocytes, facilitates their detection and quantitation [41–43]. In line with the efforts devoted to eliminating malaria transmission, molecular detection of *Plasmodium* gametocytes could help in two directions: epidemiologically for uncovering the sub-microscopic human reservoir and clinically for monitoring gametocytocidal activity of transmission-blocking anti-malarial drugs. This is particularly important since masked or invisible infectious reservoir in endemic areas is much greater than that revealed by microscopy and it maintains malaria transmission [37, 44–47].

Attempts to discover innovative, rapid techniques for the detection of gametocytes could help overcome technical difficulties encountered in determining gametocytaemia by microscopy. This becomes feasible in the field with the introduction of easy, reliable filter-paper blood collection as well as loop-mediated isothermal amplification (LAMP) technique for the detection of low-density parasite gametocytes [48–51]. The development of a reverse transcriptase-LAMP for the detection of *P. falciparum* gametocytes in clinical specimens [49] may pave the way for the innovation of rapid, simple molecular tests for detection and quantitation of sub-microscopic gametocytes for discriminating between blocking and non-blocking agents and monitoring possible changes in the activity of available gametocytocidal drugs. Other approaches to explore include concentration of gametocytes prior to amplification by PCR, PCR using a high volume (i.e., 5–15 ml) of venous blood, quantitative nucleic acid sequence based amplification, PCR with intron-spanning primers, and alternative molecular targets with multiple copies [52–55]. The search for methodologies to overcome difficulties in detecting gametocytaemia in low transmission settings should be given attention. For instance, some authors have reported the utility of examining pooled samples in malaria diagnosis from different endemic settings [56–60]. This approach needs to be investigated with regard to detecting low-density gametocytaemia in clinical trials determining the gametocytocidal activity of anti-malarial drugs. However, further research, optimization and interpretation strategies are required to test the efficiency of sample pooling in gametocyte detection in different epidemiological settings. On a broader scale, more attention should be given to explore the utility of molecular tools as a complementary approach to provide data on gametocyte load retrospectively in relation to studies on therapeutic efficacy.

One of the best available measures to address the potential of malaria parasite transmission after treatment with anti-malarial drugs may be to adopt the area-under-the-curve (AUC) of gametocyte levels combining the duration of gametocyte carriage and their density [22], although it may underestimate relationships between doses and therapeutic responses to gametocytocidal drugs in transmission-blocking studies [61]. For instance, the Four Artemisinin-Based Combinations Study Group used the AUC to assess gametocyte prevalence during follow-up of four artemisinin-based combinations in the treatment of uncomplicated malaria in seven African countries [62]. Despite the feasibility and applicability of the AUC in observational studies and clinical trials [22], its incorporation in measuring the gametocytocidal efficacy of anti-malarial drugs in routine anti-malarial drug efficacy studies is yet to be investigated. Therefore, follow-up protocols, monitoring both parasite density and gametocyte microscopic count or sub-microscopic load, have to be developed and evaluated for assessing anti-malarial drug or drug combination efficacies in different epidemiologic settings. In addition, the renewed interest in the assessment of the transmission-blocking activity of drugs and vaccines raises hope regarding the search for the best measures to evaluate their effects [61].

Apart from the determination of gametocyte carriage and gametocyte density after anti-malarial treatment, another challenge is infectivity measurement of gametocytes to vector mosquitoes. Transmissibility of infection from humans to mosquitoes is affected by a variety of host, parasite and vector factors [37]. Although it goes beyond the scope of the present opinion piece, it is noteworthy that there is no linear relationship between gametocyte density and infection rates of vector mosquitoes [63], and low-level or even sub-microscopic gametocytaemias can infect vector mosquitoes in certain settings [34–37]. In an attempt to devise tools to assess the transmissibility of infection, Joice et al. [64] recently developed and validated a method for the estimation of the relative quantities of asexual and sexual stages of *P. falciparum* that can be applied to infer maturity of gametocytes both in laboratory and in the field studies of malaria transmission.

Malaria elimination through drug-mediated transmission blocking of mature, viable male and female gametocytes to mosquitoes is a novel approach [65–67], which necessitates the adoption of appropriate strategies for assessing the transmission-blocking potential of the current and next generation of anti-malarials. The blocking potential of novel gametocytocidal agents can be determined by assessing the functional viability of drug-treated mature male and female gametocytes using *P. falciparum *dual gamete formation assay [68, 69]. Novel, high-throughput assays for assessing the functional viability of transmission stages of malaria parasites have recently been developed and validated to enable the identification of potent gametocytocidal agents that have a sterilizing effect on gametocytes, in particular on the more drug-sensitive mature male gametocytes [68, 70, 71]. Novel tools and approaches for the assessment of gametocyte parameters in drug efficacy studies will be indispensable as an increasing number of countries and sub-regions aim to eliminate malaria in the coming years.
